# How to determine life expectancy change of air pollution mortality: a time series study

**DOI:** 10.1186/1476-069X-10-25

**Published:** 2011-03-31

**Authors:** Ari Rabl, TQ Thach, PYK Chau, CM Wong

**Affiliations:** 1 ARMINES/Ecole des Mines de Paris, 60 bd. St.-Michel, 75272 Paris, France; 2 School of Public Health, The University of Hong Kong, Hong Kong SAR, PR China

## Abstract

**Background:**

Information on life expectancy (LE) change is of great concern for policy makers, as evidenced by discussions of the "harvesting" (or "mortality displacement") issue, i.e. how large an LE loss corresponds to the mortality results of time series (TS) studies. Whereas loss of LE attributable to chronic air pollution exposure can be determined from cohort studies, using life table methods, conventional TS studies have identified only deaths due to acute exposure, during the immediate past (typically the preceding one to five days), and they provide no information about the LE loss per death.

**Methods:**

We show how to obtain information on population-average LE loss by extending the observation window (largest "lag") of TS to include a sufficient number of "impact coefficients" for past exposures ("lags"). We test several methods for determining these coefficients. Once all of the coefficients have been determined, the LE change is calculated as time integral of the relative risk change after a permanent step change in exposure.

**Results:**

The method is illustrated with results for daily data of non-accidental mortality from Hong Kong for 1985 - 2005, regressed against PM_10 _and SO_2 _with observation windows up to 5 years. The majority of the coefficients is statistically significant. The magnitude of the SO_2 _coefficients is comparable to those for PM_10_. But a window of 5 years is not sufficient and the results for LE change are only a lower bound; it is consistent with what is implied by other studies of long term impacts.

**Conclusions:**

A TS analysis can determine the LE loss, but if the observation window is shorter than the relevant exposures one obtains only a lower bound.

## Background

For rational environmental policy one needs to know the life expectancy (LE) gain that can be obtained by a permanent reduction in exposure. That can be determined by means of cohort studies [1-4], in combination with life table methods for calculating the LE gain due to a change in relative risk [5-8]. The result is the total population-averaged loss due to chronic exposure. Conventional time series studies (TS), by contrast, identify only deaths due to acute exposure, during the immediate past (typically one to five days), without providing any information about the LE loss per death. For that reason the LE loss implied by TS studies of air pollution has been controversial. Before 2000 many critics contended that these deaths reflected merely a so-called "harvesting" of individuals who would have died a few days later even without pollution, an LE loss of limited relevance for rational policy decisions.

Two important papers [9,10] appear to have laid this claim to rest by extending the observation window (i.e. largest lag in the regression) up to two months and showing that the LE loss was certainly much larger than a few days. That has been confirmed by quite a few similar studies since then. However, no TS study has been able to actually calculate the LE loss due to air pollution, for two reasons: extending the observation window beyond two months encountered problems, and the explicit relation between LE loss and the coefficients of a TS was not known. In fact, the problem is complicated because there are two distinct features that are reflected in the coefficients of a TS with extended observation window: one is the lag between exposure and the resulting premature deaths, the other is the magnitude of the individual LE losses corresponding to those deaths. The present paper examines what can be learned from TS about LE loss.

Note that mortality is fundamentally different from other health outcomes because each individual will die exactly once, but can experience other endpoints, e.g. hospital stays, several times or not at all. Air pollution does not change the total number of deaths, it merely advances the date of deaths. This implies that in a TS of death rates an increase due to a pollution peak will necessarily be followed by a decrease at later times, a phenomenon that we will call "displaced deaths". Thus after a permanent increase of pollution the rate of deaths will eventually return to the original level whereas the incidence of other health endpoints will be permanently increased. Since pollution can have both immediate and delayed effects, the displaced deaths overlap the initial deaths ("direct deaths"). As shown in the Section "Direct Deaths, Displaced Deaths and Observed Deaths", these features make it impossible to ascertain the total number of air pollution deaths (here defined as any death that has been advanced by air pollution); rather, one can only find a lower bound during a specified time interval.

Since air pollution is not identifiable as cause of individual deaths, one cannot determine the LE loss by the usual method for other causes of death, namely comparing the average age of people who suffer an air pollution death with the LE of people who do not. If all air pollution deaths occurred immediately after exposure, the corresponding LE loss could be determined by the observing the decrease in death rates after the initial peak due to a pollution peak. But because of the overlap of direct and displaced deaths the individual LE loss due to an air pollution death cannot be determined.

However, as we show in the present paper, the average LE loss for the whole population (as opposed to the individuals who die because of pollution) can be determined by TS studies, at least in principle, if their observation window is long compared to the time constants of the underlying physiological processes. If the observation window is shorter (i.e. not enough lags are included), one obtains only a lower bound for the LE loss. Therefore the observation window has to be extended until one finds that any additional TS coefficients would make a negligible contribution to the total.

When all the relevant TS coefficients have been determined by regression against the concentrations, one has a dynamic model of the total (acute plus chronic) impacts of pollution on mortality. We show that when this model is applied to a permanent step change of exposure and integrated over time, one obtains the corresponding population-averaged LE change. Rearranging the resulting equation, we derive an alternative formulation whereby the LE change can be determined directly by regressing the mortality data against a combination (which we call "second differences") of the concentration data. All this requires data for total mortality and air pollution over many years; if the observation window is too short one obtains only a lower bound of the LE change.

Using TS to determine the total (acute + chronic) mortality impact of air pollution and the LE loss would be of great interest because TS data are widely available and TS studies far less costly than cohort studies. To see how well the method works in practice, we test it with daily data of total non-accidental mortality from Hong Kong for 1985 - 2005, regressed against daily PM_10 _and SO_2 _data with observation windows up to 5 yr.

## Methods

### Direct Deaths, Displaced Deaths and Observed Deaths

TS studies examine the relation between pollution and death rates D (number of deaths per unit time). D is specified in terms of deaths per short time step δt, usually taken as δt = 1 day. To begin let us consider a population that is stationary, with constant death rate D_ref _in the absence of pollution and fluctuations ΔD = D - D_ref _due to pollution(1)

**Figure 1:**
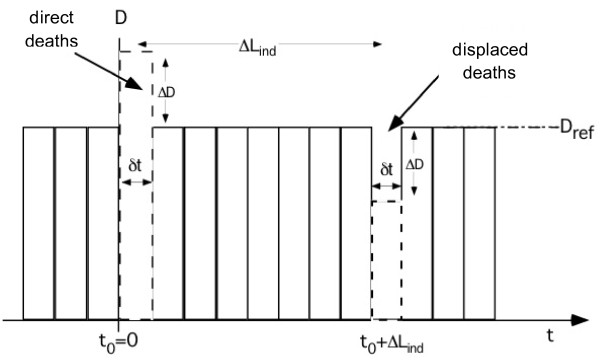
**A hypothetical situation where pollution pulse increases the death rate D of a population by ΔD between t_0 _and t_0_+δt and decreases the LE of the affected individuals by exactly ΔL_ind_**.

Now consider a hypothetical situation where a pollution pulse increases the death rate by ΔD between t_0 _and t_0_+δt and decreases LE by exactly ΔL_ind _for all affected individuals, as shown in Figure 1. Since everyone dies exactly once, the direct effect of the pulse (the "direct deaths", from t_0 _to t_0_+δt) must be followed by an equal and opposite change (the "displaced deaths", from t_0 _+ ΔL_ind _to t_0 _+ ΔL_ind_+δt). In such a situation, with a single pollution pulse, it would be easy to determine ΔL_ind_: simply look for the dip due to the displaced deaths.

**Figure 2:**
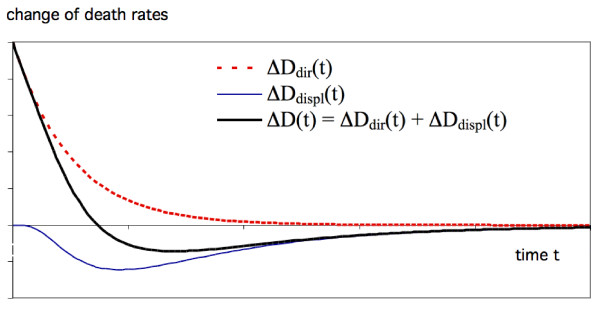
**The change in death rate due to a pollution pulse is the net result of an increase due to deaths that have been advanced, labeled ΔD_dir_(t), and a decrease due to displaced deaths, labeled ΔD_displ_(t)**. Only the total, ΔD(t) = ΔD_dir_(t) + ΔD_displ_(t), is observable. The curves are a hypothetical example with arbitrary scale.

In reality some of the pollution deaths are delayed (as evidenced by studies of mortality due to chronic exposure) and different individuals suffer different losses. For air pollution the time distributions of direct deaths ΔD_dir_(t) and displaced deaths ΔD_displ_(t) overlap, and therefore only the net change(2)

is observable, as illustrated schematically in Figure 2. The total number of direct deaths (= the time integral of ΔD_dir_(t)) equals the total number of displaced deaths (= - time integral of ΔD_displ_(t)). If all the deaths due to a pollution pulse were immediate, one could determine the average ΔL_ind, av _of the individual losses as the integral of -t ΔD_displ_(t), divided by the number of deaths due to the pulse. But whereas that may be a good assumption for heat deaths, it is certainly not for air pollution because many of the direct deaths are delayed and overlap the displaced deaths. Because of this overlap one can determine neither the number of deaths (as shown by Rabl [11]) nor the average of the individual losses.

TS studies can, however, yield information on the average LE loss ΔL_pop _of an entire population, averaged over both those who died because of pollution and those who did not. That is the quantity of interest for policy applications, and it has been calculated by the above mentioned life table methods for the cohort studies.

### Relation between Changes in Death rates and LE

To explain how one can obtain information about ΔL_pop _from fluctuations of the death rates, consider a situation where the death rate D, after being constant at D_ref_, decreases to D_ref _- ΔD during a short interval δt, thereafter resuming its old value. This means that at the end of δt a fraction |ΔD|/D_ref _of the population has lived δt longer than a population with unchanged rate. Their deaths have been postponed by δt, whenever they would otherwise have occurred; in other words, this fraction experiences an LE gain of δt. Averaged over the entire population the gain per person is(3)

There is a minus sign because ΔD is negative for a decrease of D. Thus a single dip in the death rate implies a population-averaged LE gain according to Eq.3. The instantaneous rate of LE change, averaged over the entire population, during this interval is ^a^(4)

This argument generalizes naturally to any sequence of increases and decreases of the death rate. Thus the cumulative LE change due to a sequence of changes ΔD(t) between t_1 _and t_2 _of the population-averaged relative risk is obtained by integrating Eq.4(5)

This equation for the population-averaged LE change is perfectly general, regardless of the causes of the death rate changes.

We emphasize that ΔL_pop _is the average loss over the entire population, those who are affected by pollution and those who are not. Because of the overlap between direct and displaced deaths we have found no way of determining the loss ΔL_ind _of the individuals who are affected by pollution. Even though knowledge of ΔL_ind _would be valuable for understanding the effects of pollution, for policy purposes ΔL_pop _is the most important.

**Figure 3:**
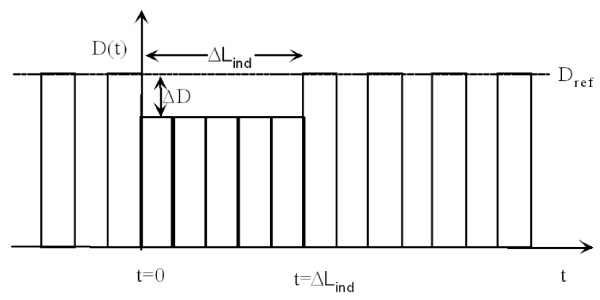
**Intervention that permanently decreases the concentration by Δc, for a hypothetical population of whom a fraction ΔD/D_ref _instantaneously gains exactly ΔL_ind_**.

Two simple examples may be instructive as illustration. The first is Figure 1 above, where a pollution peak decreases L_pop _by δt ΔD/D_ref _at t_0_, all the affected individuals losing ΔL_ind_; at t_0 _+ ΔL_ind _the population returns to its original state and L_pop _resumes its old value. As second example consider an intervention that permanently decreases the concentration of the pollutant by Δc, starting at t = 0, for a homogeneous population of whom a fraction |ΔD|/D_ref _instantaneously obtain individual gains of exactly ΔL_ind_. This is shown in Figure 3 where the death rate drops by |ΔD| at t = 0. After ΔL_ind _the death rate returns to the original level because the displaced deaths hide the decrease of the direct deaths. During each time step δt between t = 0 and t=ΔL_ind _the population gains δt |ΔD|/D_ref _and thus the total population-averaged gain is ΔL_pop _= ΔL_ind _|ΔD|/D_ref_, in agreement with Eq.5.

There have been two interventions that come close to the situation of this example. One is the regulation banning the burning of coal in Dublin after June 1990 [12,13], the other the regulation banning the use of high sulfur fuel in Hong Kong, also after June 1990 [14]. In principle ΔL_pop _for these interventions could be calculated directly by inserting the ΔD(t)/D_ref _data into Eq.5. But when we tried this approach with the mortality data for Hong Kong we encountered several problems. Above all, before inserting the data into Eq.5 one has to make sure that D(t)/D_ref _includes only changes due to the intervention. There are both seasonal variations and long term trends that must be removed from the data. In particular there has been a persistent and fairly constant long term trend of increasing LE (two to three years per decade), comparable to what has been observed in other developed countries and large enough to totally confound the intervention effect. Also, the seasonal variation is not sufficiently periodic from year to year and therefore the seasonality correction during the intervention is very uncertain. Furthermore, the reduction of SO_2 _concentrations in 1990 occurred gradually over several months rather than being a sharp step change, while other pollutants were also varying.

In the Dublin study these difficulties could be circumvented by using the mortality data of the surrounding areas as reference. Unfortunately we have no suitable reference data for Hong Kong because they are not available for that period. For these reasons the direct use of Eq.5 is problematic for Hong Kong. As an alternative we proceed in two stages. First we develop, by regression of mortality and pollution data for a long period, a dynamic model for the effect of air pollution on the ratio D(t)/D_ref_. Then we insert this model into Eq.5, evaluated for a step change in concentration, as described in the Section "Sign Reversals and Constraint for the TS Coefficients". Results for Hong Kong are presented in the Section "Regression Results".

### A Model for TS

For the choice of a suitable model for TS studies a crucial constraint comes from linearity, in view of numerous studies that have investigated the detailed form of population-level exposure-response functions for air pollution and that are providing ever more evidence that such functions are linear without threshold (see e.g. Schwartz et al. [15] for cohort studies, and Samoli et al. [16], and Daniels et al. [17] for TS). The most general linear model for the change ΔD(j) of the death rate during day j resulting from an exposure pulse of concentration c(i) during day i can be written in the form(6)

The impact of an entire exposure sequence {c(i)} of i_max _days up to day j is obtained by summation and can be written (after simple change of variables) as(7)

We shall refer to the f(i) as impact coefficients. To capture the full impact, the observation window i_max _should be as large as the sum of the relevant exposure duration and the average individual LE gains (that sum is sometimes called "cessation lag" because only after this duration do the effects of a past exposure completely disappear from a population).

Eq.7 is a straightforward and natural generalization of conventional TS; it is a distributed lag model with i_max _+ 1 lags covering the entire set of exposures that may have an impact. A variety of distributed lag models have been developed for air pollution (e.g. Zanobetti et al. [18]) to take into account some past exposures, typically only within the first month or two. In the present paper we extend the observation window to include exposures several years into the past - exposures that have been shown to be very important by several large studies of chronic mortality (e.g. Pope et al. [2]). By making the observation window sufficiently long, one can thus measure the population averaged LE loss ΔL_pop _due to pollution if the data are not too noisy.

### Comments on the Assumption of Linearity

As mentioned at the beginning of the Section "A Model for TS", linearity has been confirmed by several studies, but since those studies were carried out in countries with moderate pollution levels, it is not clear to what extent the exposure response function might be different at the much higher levels found in many cities of China, India and Africa. If the functional form of the concentration dependence is the same for all lags, the formalism of this paper could still be applied after replacing the c(i) by a function ϕ(c(i)).

Another deviation from linearity could arise from sequence effects. Eq.7 assumes that the impact coefficients need not take into account correlations between successive exposures. Such absence of correlations is assumed by practically all TS studies. In reality such correlations or sequence effects do probably exist. For instance, after a particularly heavy pollution episode the remaining number of frail individuals may be so small that the exposure during the following days entails almost no acute deaths. Accounting for such sequence effects would render the model nonlinear.

Not only would sequence effects be awkward to include in a model, but for policy applications they are irrelevant. The ultimate purpose of all this work is to provide guidance for environmental policy. It would not be appropriate to design environmental policies for a particular pollution sequence (which is unpredictable because it depends on the weather); rather they have to be applicable to any pollution sequence that may occur. Even if the real impact coefficients were to depend on the pollution sequence, for policy purposes only their average over typical sequences is relevant. Thus we conclude that the TS model of Eq.7 is indeed the appropriate choice; it is the average over typical pollution sequences. Of course, sequence effects may increase the uncertainty of any determination of the impact coefficients from a particular data set.

### Sign Reversals and Constraint for the TS Coefficients

A constraint arises from causality: the displaced deaths must occur after the direct ones. Therefore the integral of ΔD_dir_(t') from t' = 0 to t' = t must always be larger than the corresponding one of -ΔD_displ_(t'), and so the integral of the observed death rate change ΔD(t') from t' = 0 to t' = t must be non-negative for any t. This implies that the sum F(0, j) of the f(i) must satisfy the constraint of being non-negative for all j(8)

and in the limit j → ∞ it must approach zero because everybody dies exactly once. Therefore there must be at least one sign reversal in the sequence of the f(i). For the method of second differences, described below in the Section "Population-averaged LE Change from TS Data", this implies that the coefficients G(i) should not decrease with i. However, in practice the coefficients obtained by regression of data may not satisfy the constraint because of noise and confounders, unless the constraint is explicitly included in the regression model. Almost all of our results (except the two-pollutant fit for SO_2 _in the regression against second differences with adjustments within the regressions) satisfy this constraint even though we have not included the constraint in any of our regressions.

### Population-averaged LE Change from TS Data

With discrete time steps of duration T_day _= 1 day Eq.5 for the population-averaged LE gain due to a change ΔD of the death rates becomes(9)

Inserting for ΔD(j)/D_ref _the time series Eq.7 of a permanent concentration change Δc starting at t = 0 one obtains(10)

The constraint of Eq.8 implies that one gets a lower bound for ΔL_pop _when the time series is truncated, i.e. when the observation window i_max _in Eq.7 is not sufficiently long.

We had assumed a population with constant death rate in the absence of pollution, in order to simplify the explanations and the graphs. However, all the arguments hold equally if the rate D_ref _in the absence of pollution varies with time. ΔD(t) is simply the change due to pollution that is superimposed on D_ref_, and all the arguments concern only this change.

It may also be of interest to determine the LE change for different age groups. All the above arguments hold equally for a subgroup of the population that is above a specified age x_0_. Thus one can determine the LE change due to pollution for an age group above any age x_0_. Since the LE change for the entire population is the weighted average of the changes for the groups below and above age x_0_, the LE change for the group below age x_0 _can readily be calculated. Likewise the LE change for an age group between any ages x_1 _and x_2 _can also be determined.

The available evidence suggests that exposure to air pollution can affect mortality over a long period, and therefore the lags of the TS should span several years. In principle, if one had sufficiently good data, one could determine all coefficients f(i) by linear regression; but in practice most of the resulting coefficients for large lags turn out not to be statistically significant.

In order to reduce the number of coefficients and their relative standard errors one can regress ΔD against concentrations that are averaged over longer time intervals, with coefficients that are the sums of the f(i) over the corresponding intervals. With intervals of duration N_k _longer than 1 day, the f(i) c(j-i) of the TS of Eq.7 are replaced by the sums F(i_k_-N_k_+1, i_k_) of the daily coefficients f(i) from i_k_-N_k_+1 to i_k_, multiplied by the corresponding average concentrations. Thus one obtains a stairstep approximation of the f(i) for the respective intervals as(11)

The optimal choice of intervals is a matter of trial and error. An exponential time scale might be appropriate for daily data, the intervals being short immediately after a pulse and increasing to several years at long times. A possible choice might be intervals that increase exponentially into the past, for example of length 3^k ^days with k from 0 to 6: {day 1, days 2 - 4, days 5-13, days 14 - 40, days 41 - 121, days 122 - 364, days 365-1093}. The choice could be different for different pollutants since the corresponding time scales of the physiological processes are different. Another possibility is equal intervals, for instance monthly or annual.

But ΔL_pop _also can be determined directly by regression of the concentration data. Let us define(12)

where F(0, j) has been defined in Eq.8 as the sum of the f(i) from i = 0 to j. Since all the F(0, j) are positive, G(k) increases monotonically with k and its limit G(∞) is equal to LE_pop _of Eq.10. Since f(0) = F(0,0) and f(i) = F(0, i)-F(0, i-1) for i > 0, one can write the TS of Eq.7 in terms of the F(0, i) as(13)

Replacing analogously the F(0, i) by G(i)-G(i-1) for i > 0, with F(0,0) = G(0), one obtains(14)

Note that any real data series is finite and therefore we have derived this equation from the finite series of Eq.7 rather than the infinite series of Eq.10; as a consequence the terms multiplying G(i_max_) and G(i_max_-1) are different from the rest. We shall refer to the combination of concentrations [c(j-i) - 2 c(j-i-1) + c(j-i-2)] as "second differences". The main advantage of a regression with Eq.14 is to yield directly the G(i) and their confidence intervals. Another advantage is that the autocorrelation among the second differences is negligible after the first two days. The first coefficient G(0) = f(0) is the usual TS coefficient for lag 0. The last coefficient G(i_max_), multiplied by T_day _= 1 day and Δc, is an estimate of the LE change due to a permanent concentration change Δc; it is a lower bound if the observation window is not sufficiently long. Even though G(i_max_) is the coefficient of the concentration in the most distant past, all the other concentrations are taken into account in its determination because all G(i) are fitted at the same time. In fact, Eq.14 is mathematically equivalent to the ordinary TS of Eq.7; it is merely a regrouping of the independent variables of the regression.

Another method for reducing the number of coefficients is to replace the f(i) in Eq.7 or the G(i) in Eq.14 by simple functions of the lag i, for example a polynomial with coefficients to be determined by regression. But for the f(i) a polynomial fit is not suitable over such a wide range of lags because f(i) decreases rapidly during the first few days from its peak at i = 0 towards levels that are one to two orders of magnitude smaller, with slow variation; a simple polynomial of i with a small number of coefficients cannot reproduce such behavior over the entire range up to i_max_. We have not tried to see if a more complicated function might do the trick.

### Relation with LE Change from Cohort Studies

Since the usual calculation of LE loss due to air pollution is based on cohort studies such as the one of Pope et al. [2], we should comment on the relation with the method developed in the present report. The usual calculation uses the increase in age-specific mortality, as measured by cohort studies, as input into a life table calculation to yield the corresponding decrease of the LE. The calculation is static because the cohort studies that have been used as basis are in effect steady state comparisons of metropolitan areas with different exposures.

By contrast the method presented here is dynamic, being based on changes in total (rather than age-specific) mortality as measured by time series. It yields a lower bound for the LE loss which approaches the full value only to the extent that the observation window is sufficiently long to cover all relevant exposures. Another difference is that cohort studies analyze the deaths of a group of individuals, whereas the population of a TS study is in effect replenished continuously because deaths rates are normalized to a constant reference population, without identification of individuals.

In view of these differences there is no direct comparison between these two methods. The main interest of the present method lies in the far lower cost of TS studies, compared to cohort studies; another interesting aspect is that it can provide direct information on the relevant exposure window: as the observation window becomes longer than the relevant exposures, the resulting estimate of the LE loss reaches the full value that would be calculated by cohort studies and life tables. All this assumes, of course, that the data do not contain too many confounders. Thus the following part of the paper is exploratory to test the applicability of the method in practice.

## Results

### The data

In Hong Kong daily data of total non-accidental mortality and of the concentrations of NO_2_, O_3_, PM_10 _and SO_2 _are available for the period 1985 - 2005. The mortality data are for each 5 year age group, and we also have monthly population data for these age groups. As in most cities there are significant correlations between the concentration data, especially between NO_2_, O_3 _and PM_10 _(much of the NO_2 _and PM_10 _in Hong Kong is from road traffic whereas the SO_2 _comes from oil used by ships, power plants and industry). Since SO_2 _and PM_10 _are much less correlated, as shown in Table 1 we consider only regressions against these two pollutants.

**Table 1:** Correlation coefficients for the 30 day moving averages of the concentrations.

	SO_2_	PM_10_	O_3_	NO_2_
SO_2_	1			
PM_10_	-0.02	1		
O_3_	-0.22	0.33	1	
NO_2_	-0.04	0.45	0.19	1

Over the short term the concentrations of a given pollutant are highly correlated as shown in Table 2. With regard to the method of "second differences", described in Eq.14 of the Section "Population-averaged LE Change from TS Data", we note that the autocorrelation of the second differences is much smaller.

**Table 2:** Autocorrelation coefficients for concentrations and for 2nd differences of the concentrations.

SO_2 _concentrations c(day)	day	day-1	day-2	day-3	day-4	day-5
day	1.00					

day-1	0.60	1.00				

day-2	0.38	0.60	1.00			

day-3	0.31	0.38	0.60	1.00		

day-4	0.28	0.31	0.38	0.60	1.00	

day-5	0.28	0.28	0.31	0.38	0.60	1.00

SO_2 _2nd differences [c(day) - 2 c(day-1) + c(day-2)]	day	day-1	day-2	day-3	day-4	day-5

day	1.00					

day-1	-0.51	1.00				

day-2	-0.06	-0.51	1.00			

day-3	0.07	-0.06	-0.51	1.00		

day-4	-0.01	0.06	-0.06	-0.51	1.00	

day-5	0.01	-0.01	0.06	-0.06	-0.51	1.00

PM_10 _concentrations c(day)	day	day-1	day-2	day-3	day-4	day-5

day	1.00					

day-1	0.82	1.00				

day-2	0.65	0.82	1.00			

day-3	0.53	0.65	0.82	1.00		

day-4	0.46	0.53	0.65	0.82	1.00	

day-5	0.41	0.46	0.53	0.65	0.82	1.00

PM_10 _2nd differences [c(day) - 2 c(day-1) + c(day-2)]	day	day-1	day-2	day-3	day-4	day-5

day	1.00					

day-1	-0.46	1.00				

day-2	-0.04	-0.46	1.00			

day-3	-0.03	-0.04	-0.46	1.00		

day-4	0.02	-0.03	-0.04	-0.46	1.00	

day-5	-0.01	0.02	-0.03	-0.04	-0.46	1.00

**Figure 4:**
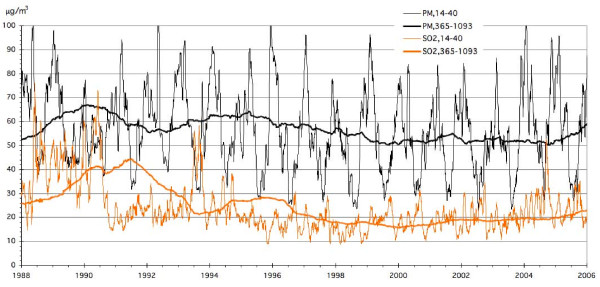
**Concentration data for SO_2 _and PM_10_, shown as moving averages during the periods 14-40 days and 365-1093 days before the current date**.

The concentration data for SO_2 _and PM_10 _are shown in Figure 4. as moving averages during the periods 14-40 days and 365-1093 days before the current date. We do not show the daily data because the graph would be unreadable but the series in this figure suffice to show both short term and long term variations. The intervals correspond to the periods in the Section "Results with adjustments before the regressions against pollution". The SO_2 _intervention occurred July 1990. The average concentration for the entire period is 56.7 μg/m^3 ^for PM_10 _and 24.8 μg/m^3 ^for SO_2_.

### Preparation of the data

Since the formalism of the Section "Methods" assumes a stationary population, one has to adjust the mortality data so that they correspond to an equivalent stationary population. We have done this in several steps, the first being an adjustment for the age structure. A plot of the age distribution shows that the age structure of Hong Kong has been changing during this period and that the time average of the distributions is not very different from the standard age profile of the World Health Organization [19]. Therefore we choose this age profile to calculate age-standardized mortality rates.

Additional adjustments are needed for temperature, humidity, seasonal patterns and long term trends. For these adjustments we have taken two different approaches. The first, described in the Section "Adjustments before the regressions against pollution", makes the adjustments before the regressions against pollution. The second, in the Section "Adjustments within the regressions against pollution", includes the adjustments within the regressions.

#### Adjustments before the regressions against pollution

**Figure 5:**
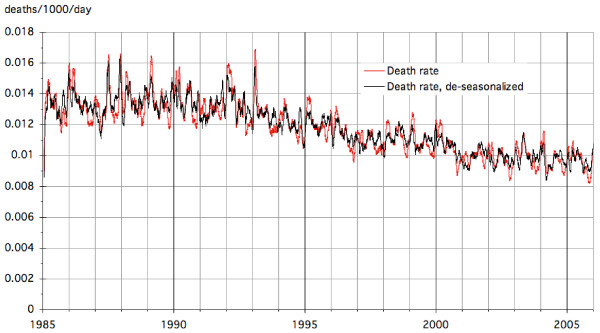
**Death rates before and after adjustment for seasonality**.

With this approach we begin with nonparametric smoothing to correct for temperature T and humidity H, to obtain equivalent death rates at the average T and H. Then we de-seasonalize the mortality rates because they display fairly systematic seasonal variations, even after correction for T and H. To de-seasonalize, we have calculated a standard seasonal death rate profile, normalized to an average of unity, and then divided the death rates by this profile. We calculated the standard seasonal profile by first replacing the data by their 30 day moving averages, then averaging for each day of the year the values for the respective day of each of the 20 years, and finally normalizing the profile to an average of unity (we have also tried moving averages for periods shorter than 30 days but found that shorter averaging periods yielded too many irregular daily variations that stemmed from the limited number of data and did not reflect the true seasonal trend). The death rates before and after this adjustment for seasonality are plotted in Figure 5 as 30-day moving averages. The fluctuations are significantly reduced by this adjustment but remain large. We have used the same method for de-seasonalizing the concentrations.

A final step removes the long term trend because Hong Kong, like most industrialized countries, has been enjoying a steady increase in LE, averaging about two to three years per decade. We therefore calculate the reference death rate D_ref _as moving average of the D(t) over the observation window. For example, with a choice of 7 intervals of durations 3^0 ^to 3^6 ^days, the total observation window is the sum of these 7 intervals, 1093 days, and D_ref_(t) is the average of the D(t') from t' = t to t-1092. This step removes the long term trend of mortality from D(t)/D_ref_(t) since numerator and denominator have the same trend. The result looks like the de-seasonalized series in Figure 5 but without the declining trend after 1993. For our regressions we used the Statistics Add-on of Mathematica and we checked some of the results with the software R [20].

#### Adjustments within the regressions against pollution

As an alternative approach we have made the adjustments for seasonality, long term trend, temperature and humidity within the regressions. For this we have decomposed the death rates into three components: seasonality trends, long-term trends and residuals, following a similar approach proposed by Schwartz [9] and using the STL algorithm of Cleveland et al. [21]. We then adjust the de-seasonalized death rates for trends, temperature and humidity by means of regression. We applied the non-parametric LOESS smoothing to each regressor with window span = 0.5. We obtain the reference death rate D_ref _by calculating the adjusted means of D(t) evaluating temperature and humidity at their mean levels and regress ΔD(t)/D_ref _against the deviations of the concentrations from their moving average during the observation window. For these regressions we used the software R.

### Regression Results

#### Results with adjustments before the regressions against pollution

**Figure 6:**
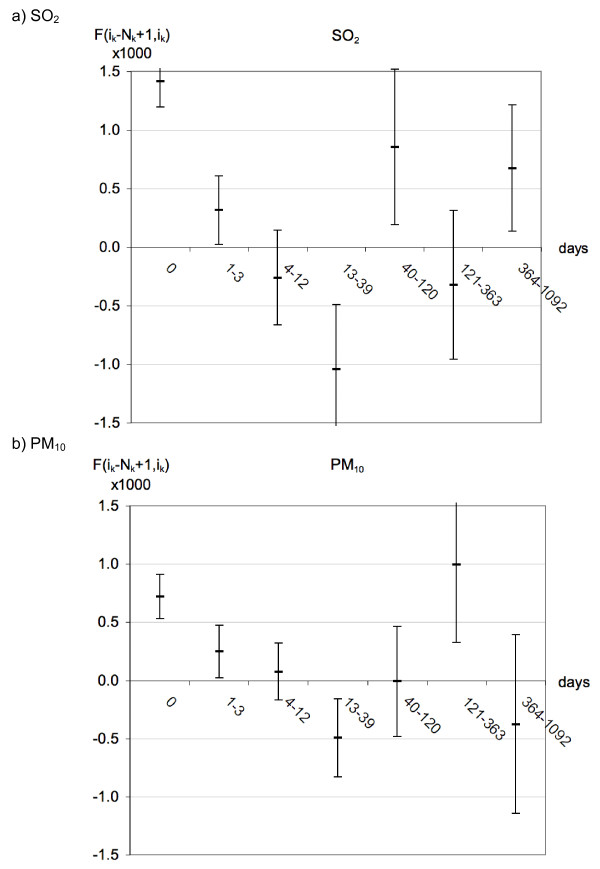
**The coefficients F(i_k_-N_k_+1, i_k_) for fits (with adjustments before the regressions against pollutants) with 7 intervals of lengths N_k _= 3^k ^days, k = 0 to 6, for SO_2 _(part a) and PM_10 _(part b)**. The CI are symmetric. Units of F are [μg/m^3^]^-1^.

For the regressions against averaged concentrations (TS of Eq.7 but with averages over intervals according to Eq.11), we show results for 7 intervals of length N_k _= 3^k ^days (k = 0 to 6), for single pollutant regressions with PM_10 _and SO_2_. The intervals are {day 0, days 1-3, days 4-12, days 13-39, days 40-120, days 121-363, days 364-1092}. The coefficients F(i_k_-N_k_+1, i_k_) for these intervals are shown in Figure 6.

The first coefficient, F(0,0), equals the coefficient f(0) of the TS since the first interval is one day; it can be compared with the results of conventional TS. For SO_2 _we have f(0) = 0.0014 and for PM_10 _0.00072. Note that the units of F are [μg/m^3^]^-1^; since Figure 6 shows F × 1000 we have in effect converted to the more customary % per 10 μg/m^3^. The LE change is 1.34 days/(μg/m^3^) for SO_2 _and 1.31 days/(μg/m^3^) for PM_10_. These numbers are lower bounds since the observation window is not long enough.

**Figure 7:**
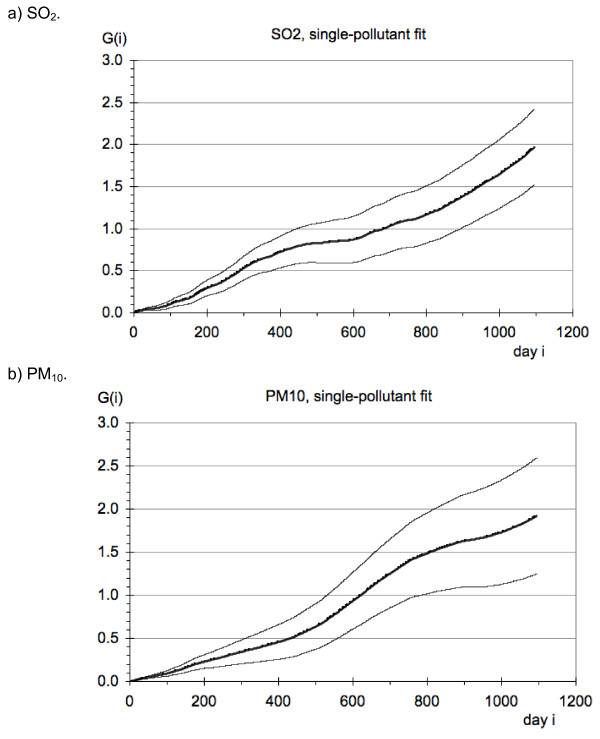
**Results for the G(i) coefficient of the regressions against the second differences, Eq.14, single pollutant fits with adjustments before the regressions**. The units of G(i) are [μg/m^3^]^-1^.

Results for the G(i) coefficients of the regressions against the second differences, Eq.14, are shown in Figure 7 for single-pollutant regressions only, with observation window 3 yr, i_max _= 1096 days. The G(i) in our plots are smooth (apart from being stepwise from one i to the next) even though all i_max _terms were estimated together, without any smoothing or constraint. The first coefficient, G(0), equals the coefficient f(0) of the TS. It is 0.67% per 10 μg/m^3 ^for PM_10 _and 1.4% per 10 μg/m^3 ^for SO_2_. The last coefficient, G(1096) in this case, multiplied by T_day _= 1 day, is a lower bound estimate of the LE change; it is 1.92 days/(μg/m^3^) for PM_10 _and 1.97 days/(μg/m^3^) for SO_2_. Within the 4 Gb of RAM limitation of our computers we have been able to extend the window to 5 years. In the graphs (not shown) the G(i) continue to increase without sign of leveling off, and the lower bound estimate of the LE change is 3.58 days/(μg/m^3^) for PM_10 _and 3.80 days/(μg/m^3^) for SO_2 _for a window of 5 yr.

#### Results with adjustments within the regressions against pollution

**Figure 8:**
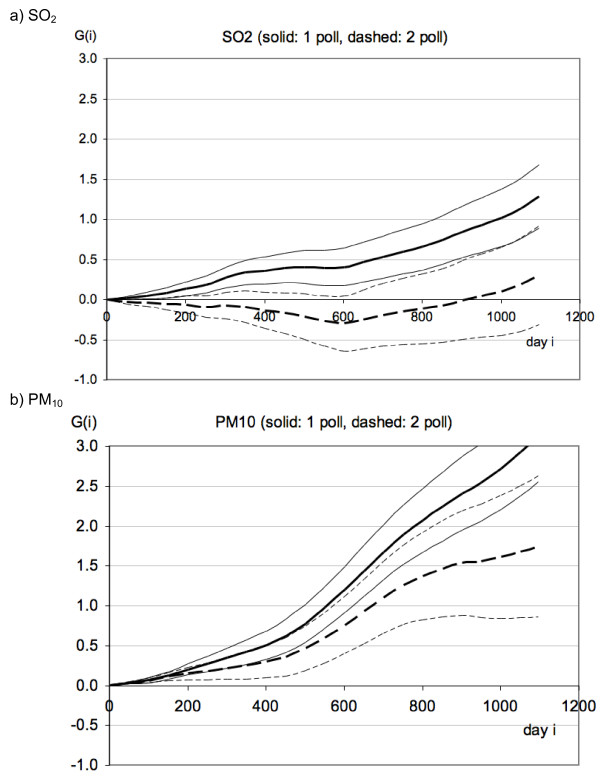
**Results for the G(i) coefficient of the regressions against the second differences (with adjustments within the regressions), Eq.14, for fits with one and two pollutants (SO_2 _and PM_10_)**. The units of G(i) are [μg/m^3^]^-1^.

For the approach with adjustments within the regression we only have fits for the G(i), shown in Figure 8. For SO_2 _the first coefficient, G(0), is 0.75% per 10 μg/m^3 ^for the single pollutant fit and 0.73% per 10 μg/m^3 ^for the fit with two pollutants; the last coefficient, G(1096) in this case, is 1.28 days/(μg/m^3^) for the single pollutant fit and 0.3 days/(μg/m^3^) for the fit with two pollutants. For PM_10 _the first coefficient, G(0), is 0.21% per 10 μg/m^3 ^for the single pollutant fit and 0.19% per 10 μg/m^3 ^for the fit with two pollutants; the last coefficient, G(1096) in this case, multiplied by T_day _= 1 day, is 3.14 days/(μg/m^3^) for the single pollutant fit and 1.74 days/(μg/m^3^) for the fit with two pollutants.

#### Sensitivity analyses

To answer possible concerns about the adjustments for confounders, we have performed several sensitivity studies. The first of these addresses the adjustment for temperature and humidity (T and H). Whereas the main analyses adjusted only for temperature and humidity of the current day, we also tested variants where we extended this adjustment to cover longer periods, up to 30 days, the adjustments for T and H and for seasonal and long term trends being done within the regression. Even with 30 days the change of the coefficient f(0) was only as large as the standard error, and the LE change G(1096) changed by only 3%.

Since the models we have used for seasonal variations are not perfect and in any case the true seasonality varies from year to year, some concern about seasonal confounding remains. As a simple test of the sensitivity to seasonal confounding, we have repeated some of the regressions by adding an artificial sinusoidal variation to the death rates, comparable in magnitude to the observed seasonal variation. Specifically we have taken the death rates (as adjusted for seasonality and trend before the regression) and multiplied them by a factor 1 + 0.05 × cos(2 π (day + day_0_)/365), choosing different values, 0, 91, 182 and 273, for day_0_. Then we have repeated the 2^nd ^difference regressions (as described in the Section "Results with adjustments before the regressions against pollution") using these modified rates. The results for the f(0) coefficient change by less than the standard error. For the LE change the results vary by +-28% for SO_2 _and by +-22% for PM_10_, depending on the choice of day_0_. Such variation is a bit larger than the standard error but still within the 95% confidence interval. As a more rigorous test one could vary the multiplying factor from year to year, but this simple test already indicates that seasonal confounding can be appreciable but does not invalidate the approach.

## Discussion

### Comparisons with other short term studies

Table 3 summarizes our results for the impact coefficient f(0) ("lag 0") which has also been measured in most of the conventional TS studies of air pollution mortality. Among the countless studies of that type we cite Stieb et al. [22] and Daniels et al. [23]. The one by Stieb et al. is appropriate because it is a large worldwide meta-analysis (109 studies) and it included SO_2_; their random effects pooled estimate of excess all-cause mortality, for single-pollutant models is shown in Table 3. Daniels et al. reported the results of the National Morbidity, Mortality and Air Pollution Study (NMMAPS) for the 20 largest cities in the USA, as found with the log-linear model for the average of the current and previous day. Note that the TS studies prior to 2003 suffered from a defect due to the default algorithm used for GAM (generalized additive models) which tended to overestimate the effect by perhaps a third; the present paper is not affected because it does not use that algorithm. Table 3 compares our results for Hong Kong with these two studies. The results are not out of line, considering that the confidence intervals of meta-analyses and large multi-city studies are much smaller than the range of values found in individual studies. The present results are also consistent with a Hong Kong study [24] that found 0.87% (95% CI 0.38, 1.36) for SO_2_, and 0.53% (95% CI 0.26, 0.81) for PM_10_.

**Table 3:** Comparison of our results for impact coefficient f(0) with conventional TS studies.

Study	PM_10_	SO_2_	Comments
This paper: regressions of concentrations, TS of Eq.7, but with sums over variable intervals Eq.11	0.72 (0.53 to 0.91)	1.42 (1.20 to 1.64)	Single pollutant regressions with 7 intervals of length 3^k ^days, k = 0 to 6, adjustments before regression
This paper: regressions of second differences of concentrations, Eq.14	0.67 (0.4 to 0.93)	1.40 (1.1 to 1.7)	Single pollutant regressions with 1096 coefficients, adjustments before regression
This paper: regressions of second differences of concentrations, Eq.14	0.21 (-0.03 to 0.46)	0.75 (0.49 to 1.0)	Single pollutant regressions with 1096 coefficients, adjustments within regression
Stieb et al. [22]	0.64 (0.48 to 0.77)	0.36 (0.28 to 0.48)	worldwide meta-analysis (109 studies)
Daniels et al. [17]	0.28 (0.16 to 0.41)		NMMAPS of the 20 largest cities in the USA

Units are % excess mortality risks per 10 μg/m^3 ^for PM_10 _and SO_2_. Confidence intervals in parentheses.

### Comparisons with other long term studies

Table 4 shows a summary of our results for LE change and a comparison with other long term studies. Here the recent study of long term mortality due to black smoke and SO_2 _by Elliott et al. [25] is most relevant. These authors measured in effect the coefficients F_0-4 yr_, F_0-8 yr_, F_0-12 yr _and F_0-16 yr_, corresponding to the exposure windows 0-4, 0-8, 0-12 and 0-16 years. These F's are the sums of the impact coefficients from 0 to 4, 8, 12, and 16 years, respectively. The results of Elliott et al. for all cause mortality are reproduced in Table 5. The fact that the F decrease with length of the window indicates that the impact coefficients are negative after 4 years (due to displaced deaths) and are still different from zero between 12 and 16 years, for black smoke and for SO_2_. A calculation according to Eq.10 (but with time step 4 years instead of 1 day) yields an LE loss of 39 days per 10 μg/m^3 ^increase of black smoke and 48 days per 10 μg/m^3 ^increase of SO_2_.

**Table 4:** Comparison of LE losses from long term studies, in days per 10 μg/m^3^.

Study	PM_10_	SO_2_	Comments
This paper: regressions of concentrations, TS of Eq.7, but with sums over variable intervals Eq.11	13.1	13.4	Single pollutant regressions with 7 intervals of length 3^k ^days, k = 0 to 6, adjustments before regression; observation window 3 yr
This paper: regressions of second differences of concentrations, Eq.14	19.2 (12.5 to 25.9)	19.7 (15.2 to 24.2)	Single pollutant regressions with 1096 coefficients G(i), adjustments before regression; observation window 3 yr
This paper: regressions of second differences of concentrations, Eq.14	35.8 (21.8 to 49.8)	38.0 (27.4 to 48.6)	Single pollutant regressions with 1825 coefficients G(i), adjustments before regression; observation window 5 yr
This paper: regressions of second differences of concentrations, Eq. 14	31.4 (25.6 to 37.2)	12.8 (8.9 to 16.8)	Single pollutant regressions with 1096 coefficients, adjustments within regression; observation window 3 yr
Elliott et al. [25]	39 × conversion factor black smoke/PM_10_	48	Eq.10 with numbers of Table 5 (but with time step 4 years instead of 1 day); observation window 16 yr
Cohort studies, in particular Pope et al. [2], with calculation of LE loss by Rabl [11].	90 ^a^	110 ^b^	Mean concentration 28.8 μg/m^3 ^for PM_10 _and 17.8 μg/m^3 ^for SO_2 _in 1982-98

Note that the estimates of this paper are only a lower bound because the G(i) have not yet leveled off.

a) taking RR = 1.06 for 10 μg/m^3 ^PM_2.5 _from Table 2 of Pope et al. [2] and assuming a factor 0.6 for the conversion from PM_2.5 _to PM_10_

b) rough estimate, reading RR from Fig.5 of Pope et al. [2]

**Table 5:** Results of Elliott et al. [25] for all-cause mortality by exposure window (adjusted for deprivation and urban/rural classification), as extracted from their Table 3.

Exposure window (years)	% excess relative risk (95% credible intervals)
black smoke (% per 10 μg/m^3^)	
0-4	1.3 (1.0 to 1.6)
0-8	0.7 (0.6 to 0.9)
0-12	0.5 (0.5 to 0.6)
0-16	0.4 (0.4 to 0.4)
SO_2 _(per 10 ppb)	
0-4	4.2 (3.6 to 4.8)
0-8	2.5 (2.2 to 2.7)
0-12	1.6 (1.4 to 1.7)
0-16	1.0 (0.9 to 1.1)

The LE loss for cohort studies has been calculated by Rabl [26] and Miller and Hurley [8], based on Pope et al. [2]. The results depend of course on the value of the risk increase and they vary somewhat with the population to which it is applied. Typical results are around 90 days per 10 μg/m^3 ^of PM_10 _(assuming a factor 0.6 for the conversion from PM_2.5 _to PM_10_). Pope et al. also find a significant association of all-cause mortality with SO_2 _as shown in Figure 5 of their paper; from that we obtain a rough estimate of 110 days per 10 μg/m^3 ^of SO_2_. Our results for LE change are lower bounds and consistent with what is implied by the long term studies of Pope et al. and Elliott et al. The concentration levels for PM_10 _in Hong Kong are about twice as high as for the study of Pope et al. and for SO_2 _they are about 40% higher, so the mortality impacts should not be too different even if the exposure response-functions were to become nonlinear at higher concentrations.

There are also parallels with the cohort study of Schwartz et al. [15]. These authors carried out an extended follow-up of the Harvard Six Cities cohort study [1]. One of the features of that work is an analysis by exposure window ("lag"), with exposure intervals of 1 to 5 years preceding death. Only associations with PM_2.5 _were reported. However, whereas Schwartz et al. found that the excess risk decreases rapidly from one year to the next and is negligible beyond the 2^nd ^year, we find that past exposure is significant for at least three years. This result of Schwartz et al. is very different from that of another recent cohort study, by Puett et al. [27], which finds that the coefficients for all-cause mortality due to PM_2.5 _increase for exposures in previous years up to 3 years and begin to decline only slightly for the fourth year (the longest exposure considered in that study). Krewski et al. [28] tried to find an answer by analyzing exposure windows of 0-5 yr, 6-10 yr and 11-15 yr for the ACS cohort but were unable to draw firm conclusions about the relevant exposure windows.

Determining the relevant exposure window for air pollution seems to be very difficult because one needs good data with sufficient exposure contrast over a long time period. Because of many commonalities (combustion particles, cardiopulmonary disease and lung cancer) between air pollution and smoking, it is of interest to note that studies of smoking cessation suggest that the relevant exposure window may be more than a decade, see in particular the data in Figure 4 of the extraordinary study of Doll et al. [29]. We conclude that the issue of the relevant exposure window does not yet seem to be settled.

As so often with TS, the coefficients can change more than they should when another pollutant is added to a single pollutant model. That is due to the unfortunate fact that the concentrations of different pollutants tend to be correlated. Even for a single pollutant the concentrations during different time intervals are correlated and so it is not surprising that the impact coefficients can turn out fairly different between regressions with different interval choices.

The LE losses of the present paper should be smaller than the ones implied by Elliott et al. and Pope et al. since our observation window is only 3 years whereas these latter studies have windows of 16 years. In particular the results of Elliott et al. in Table 5 imply that the impact coefficients are still non-zero between 12 and 16 years. Thus with a window of 3 years we capture only a small portion of the full LE loss.

## Conclusions

We had two objectives for this paper: (i) to develop a method for determining LE change from TS studies and (ii) to test this method with data for Hong Kong. Our TS model, Eq.7, is a natural generalization of conventional TS to account for long term exposures by including a large number of lags. Eq.10 for the population-average LE change attributable to air pollution follows logically but it yields only a lower bound which increases with the length of the observation window, i.e. the number of lags included in the TS. We present two ways to implement the approach: one involves averaging over extended time intervals, the other involves a change of independent variables to what we call "second differences". Thus the LE change can be determined, at least in principle, if one has sufficiently good data for a sufficiently long period.

To test the method we had a data series of 20 years, and we could have extended the window to about ten years. However, we also wanted to include the period of the SO_2 _intervention of July 1990 because it involved the largest change in SO_2 _concentrations. Since the data started only in January 1985, we limited the window to three years (if the TS starts at time t_0_, the pollution data must be available for the entire length of the observation window before t_0_).

We started out by regressing the death rates against concentrations, as is customary in TS studies. But we found regressions against second differences of the concentrations, Eq.14, preferable because they yield directly the LE change as well as its confidence interval, whereas we do not know the CI for the LE change calculated from the f(i) coefficients of the customary TS. In addition the second difference regressions yield directly the first coefficient f(0) ("lag 0") of the customary TS, together with its CI.

Like Elliott et al., we found a very significant association of mortality with SO_2_, of the same order of magnitude as the one for PM_10_, even though many epidemiologists doubt that SO_2 _could have such an effect. Conceivably this effect could be due to transition metals, in particular Ni and V, that are emitted by the dominant SO_2 _sources, namely combustion of oil or coal. Such metals have been identified in some studies as possible agents that increase the toxicity of ambient PM (e.g. Lippmann et al. [30]). In Hong Kong the SO_2 _comes mainly from the combustion of heavy fuel oil which contains significant amounts of such metals. The ratios Ni/SO_2 _and V/SO_2 _are extremely variable from site to site because the trace metal content of coal and oil can vary by an order of magnitude between different sources; additional differences can arise from the pollution control technologies used. Such variability could explain the lack of consistency between different studies of health impacts of SO_2_.

This second part of our paper has been frankly exploratory. The results are promising and entirely consistent with short term and long term studies in the literature. But even our longest observation window of five years is not sufficient and the results for LE change are only a lower bound.

There are quite a few different possibilities for implementing the method and we are not sure which is best (especially the adjustment for seasonality, long term trend, temperature and humidity). We are uneasy about differences between different regressions, for instance Figure 7 and Figure 8. Also, we do not know how far the observation window can be extended in view of limited exposure contrast. A systematic exploration of these issues is desirable but beyond the scope of the present paper. We have just been awarded a new research contract to carry out a range of validation studies and we will analyze NMMAPS data in addition to data for Hong Kong, now extended to 2010.

Our calculation of LE loss can also be applied to the analysis of other burdens that are not identifiable as cause of individual deaths, for instance heat waves. However, since it assumes that the burden of concern (e.g. air pollution) does not change the number of deaths, it is not appropriate for mortality due to specific causes (e.g. respiratory mortality) if the burden can change the proportions of specific identified causes. For example, a change in air pollution could shift some deaths from respiratory to cardio-vascular and therefore one cannot calculate the LE loss due to respiratory air pollution mortality. Accidents, on the other hand, are not affected by the usual air pollution and therefore we have used the method for data of non-accidental mortality.

Finally we point out that the phenomenon of displaced deaths in a TS study does not apply to endpoints such as hospital visits that an individual can experience several times or not at all. Such endpoints show up very differently in TS of interventions. After a permanent pollution reduction the mortality will decrease only temporarily and return to the initial rate when the postponed deaths finally occur, whereas the incidence of non-mortality endpoints will be permanently reduced.

## Abbreviations

c(i): pollutant concentration on day i; D: death rate (number of deaths per unit time); D_ref_: reference death rate; ΔD: D - D_ref_; ΔL_ind_: LE change of individuals; ΔL_pop_: population-average LE change; ΔD_dir_(t): direct deaths (of death rate after pollution pulse); ΔD_displ_(t): displaced deaths (of death rate after pollution pulse); δt: time step (usually 1 day); Eq.: equation;Fig.: figure; f(j): impact coefficients; F(0, j): sum of the f(i) from i = 0 to j; G(k): sum of the F(0, j) from j = 0 to k; LE: life expectancy; RR: relative risk; T_day_: 1 day; TS: time series.

## Competing interests

The authors declare that they have no competing interests.

## Authors' contributions

AR developed the method for estimating LE change and performed some of the regressions; TQT developed the method of second differences and performed some of the regressions; PYKC collected and extracted data, CMW initiated the idea and partially inputted on the development of the methods. All authors read and approved the final manuscript.

## References

[B1] Dockery DW Pope CA III Xu Xiping Spengler JD Ware JH Fay ME Ferris BG Speizer FE An association between air pollution and mortality in six US cities New England J of Medicine 1993 329 1753 1759 10.1056/NEJM199312093292401 8179653

[B2] Pope CA Burnett RT Thun MJ Calle EE Krewski D Ito K Thurston GD Lung cancer, cardiopulmonary mortality, and long term exposure to fine particulate air pollution J Amer Med Assoc 2002 287 9 1132 1141 10.1001/jama.287.9.1132 PMC4037163 11879110

[B3] Laden F Schwartz J Speizer FE Dockery DW Reduction in fine particulate air pollution and mortality: extended follow-up of the Harvard Six Cities Study Am J Respir Crit Care Med 2006 173 6 667 672 10.1164/rccm.200503-443OC 16424447 PMC2662950

[B4] Hoek G Brunekreef B Goldbohm S Fischer P van den Brandt PA Association between mortality and indicators of traffic-related air pollution in the Netherlands: a cohort study Lancet 2002 360 October 19 1203 1209 10.1016/S0140-6736(02)11280-3 12401246

[B5] Brunekreef B Air pollution and life expectancy: is there a relation? Occupational and Environmental Medicine 1997 54 781 784 10.1136/oem.54.11.781 9538349 PMC1128948

[B6] Rabl A Mortality risks of air pollution: the role of exposure-response functions Journal of Hazardous Materials 1998 61 91 98 10.1016/S0304-3894(98)00112-5

[B7] Leksell L Rabl A Air Pollution and Mortality: Quantification and Valuation of Years of Life Lost Risk Analysis 2001 21 5 843 857 10.1111/0272-4332.215156 11798121

[B8] Miller BG Hurley JF Life table methods for quantitative impact assessments in chronic mortality J Epidemiology and Community Health 2003 57 200 206 10.1136/jech.57.3.200 PMC1732393 12594196

[B9] Schwartz J Mortality Displacement and Long-term Exposure Effects Related to Air Pollution and Mortality 2000 http://www.healtheffects.org/Section 4 of Samet JM, F Dominici, SL Zeger, J Schwartz, W Dockery. **National Morbidity, Mortality, and Air Pollution Study, Part I: Methods and Methodologic Issues**. Research report 94, Part 1, 2000. The Health Effects Institute, Cambridge MA

[B10] Zeger SL Dominici F Samet JM Mortality Displacement-Resistant Estimates of Air Pollution: Effects on Mortality http://www.healtheffects.org/Section 3 of Samet JM, F Dominici, SL Zeger, J Schwartz, W Dockery. **National Morbidity, Mortality, and Air Pollution Study, Part I: Methods and Methodologic Issues**. Research report 94, Part 1, 2000. The Health Effects Institute, Cambridge MA 11098531

[B11] Rabl A Analysis of air pollution mortality in terms of life expectancy changes: relation between time series, intervention and cohort studies Environmental Health: A Global Access Science Source 2006 5 1 http://www.ehjournal.net/content/5/1/1 10.1186/1476-069X-5-1 16451722 PMC1373624

[B12] Clancy L Goodman P Sinclair H Dockery DW Effect of air-pollution control on death rates in Dublin, Ireland: an intervention study Lancet 2002 360 1210 4 10.1016/S0140-6736(02)11281-5 12401247

[B13] Goodman PG Dockery DW Luke Clancy L Cause-Specific Mortality and the Extended Effects of Particulate Pollution and Temperature Exposure Environmental Health Perspectives 2004 112 2 179 185 10.1289/ehp.6451 14754572 PMC1241827

[B14] Hedley AJ Wong CM Thach TQ Ma S Lam TH Anderson HR Cardiorespiratory and all-cause mortality after restrictions on sulphur content of fuel in Hong Kong: an intervention study Lancet 2002 360 1646 52 10.1016/S0140-6736(02)11612-6 12457788

[B15] Schwartz J Coull B Laden F Ryan L The Effect of Dose and Timing of Dose on the Association between Airborne Particles and Survival Environmental Health Perspectives 2008 116 1 64 69 10.1289/ehp.9955 18197301 PMC2199297

[B16] Samoli E Analitis A Touloumi G Schwartz J Anderson HR Sunyer J Bisanti L Zmirou D Vonk JM Pekkanen J Goodman P Paldy A Schindler C Katsouyanni K Estimating the Exposure-Response Relationships between Particulate Matter and Mortality within the APHEA Multicity Project Environmental Health Perspectives 2005 113 1 88 95 10.1289/ehp.7387 15626653 PMC1253715

[B17] Daniels MJ Dominici F Samet JM Zeger SL Estimating particulate matter-mortality dose-response curves and threshold levels: an analysis of daily time-series for the 20 largest US cities Am J Epidemiol 2000 152 5 397 406 See also Comment in: Am J Epidemiol., **152(5):**407-12 10.1093/aje/152.5.397 10981451

[B18] Zanobetti A Wand MP Schwartz J Ryan LM Generalized additive distributed lag models: quantifying mortality displacement Biostatistics 2000 1 3 279 292 10.1093/biostatistics/1.3.279 12933509

[B19] WHO Omar B Ahmad, Cynthia Boschi-Pinto, Alan D Lopez, Christopher JL Murray, Rafael Lozano, Mie Inoue Age Standardization of Rates: a New WHO Standard GPE Discussion Paper Series: No.31, EIP/GPE/EBD 2000 World Health Organization

[B20] R Development Core Team R: A Language and Environment for Statistical Computing R Foundation for Statistical Computing, Vienna, Austria

[B21] Cleveland RB Cleveland WS McRae JE Seasonal-trend decomposition procedure based on LOESS J Offic Stat 1990 6 3 73

[B22] Stieb DM Judek S Burnett RT Meta-Analysis of Time-Series Studies of Air Pollution and Mortality: Effects of Gases and Particles and the Influence of Cause of Death, Age, and Season J Air & Waste Manage Assoc 2002 52 April 470 484 10.1080/10473289.2002.10470794 12002192

[B23] Daniels MJ Dominici F Zeger SL Jonathan M Samet JM The National Morbidity, Mortality, and Air Pollution Study, Part III: PM10 Concentration-Response Curves and Thresholds for the 20 Largest US Cities 2004 Health Effects Institute Research Report Number 94, Part III 15457982

[B24] Wong CM Vichit-Vadakan N Kan H Qian Z Public Health and Air Pollution in Asia (PAPA): a multicity study of short-term effects of air pollution on mortality Environ Health Perspect 2008 116 9 1189 94 10.1289/ehp.10850 18795163 PMC2535622

[B25] Elliott P Shaddick G Wakefield JC de Hoogh C Briggs DJ Long-term associations of outdoor air pollution with mortality in Great Britain Thorax 2007 62 1088 1094 10.1136/thx.2006.076851 17666438 PMC2094283

[B26] Rabl A Interpretation of Air Pollution Mortality: Number of Deaths or Years of Life Lost? Journal of the Air & Waste Management Association 2003 53 1 41 50 10.1080/10473289.2003.10466118 12568252

[B27] Puett RC Hart JE Yanosky JD Paciorek C Schwartz J Suh H Speizer FE Laden F Chronic Fine and Coarse Particulate Exposure, Mortality, and Coronary Heart Disease in the Nurses' Health Study Environmental Health Perspectives 2009 117 11 10.1289/ehp.0900572 PMC2801178 20049120

[B28] Krewski D Jerrett M Burnett RT Ma R Hughes E Shi Y Turner MC Pope CA Thurston G Calle EE Michael J Thun MJ Extended Follow-Up and Spatial Analysis of the American Cancer Society Study Linking Particulate Air Pollution and Mortality HEI Report 140 2009 Health Effects Institute, Boston, Massachusetts 19627030

[B29] Doll R Peto R Boreham J Sutherland I Mortality in relation to smoking: 50 years' observations on male British doctors British Medical Journal 2004 328 7455 1519 1527 10.1136/bmj.38142.554479.AE 15213107 PMC437139

[B30] Lippmann M Ito K Maciejczyk P Chen L-C Cardiovascular Effects of Nickel in Ambient Air Environmental Health Perspectives 2006 114 11 1662 1669 17107850 10.1289/ehp.9150 PMC1665439

